# Management of adults with Alagille syndrome

**DOI:** 10.1007/s12072-023-10578-x

**Published:** 2023-08-16

**Authors:** Mohammed D. Ayoub, Ahmad A. Bakhsh, Shannon M. Vandriel, Verena Keitel, Binita M. Kamath

**Affiliations:** 1 Department of Pediatrics, Faculty of Medicine, Rabigh Branch, King Abdulaziz University, Jeddah, Saudi Arabia; 2 Division of Gastroenterology, Hepatology, and Nutrition, The Hospital for Sick Children, University of Toronto, 555 University Ave, Toronto, ON M5G 1X8 Canada; 3 Department of Pediatrics, Faculty of Medicine, University of Jeddah, Jeddah, Saudi Arabia; 4 Department of Gastroenterology, Hepatology and Infectious Diseases, Faculty of Medicine, Otto Von Guericke University Magdeburg, Magdeburg, Germany

**Keywords:** Bile duct paucity, *JAG1*, *NOTCH2*, Cholestatic liver disease, Pruritus, Maralixibat, Portal hypertension, Liver transplant, Regenerative nodules, Hepatocellular carcinoma

## Abstract

Alagille syndrome (ALGS) is a complex rare genetic disorder that involves multiple organ systems and is historically regarded as a disease of childhood. Since it is inherited in an autosomal dominant manner in 40% of patients, it carries many implications for genetic counselling of patients and screening of family members. In addition, the considerable variable expression and absence of a clear genotype–phenotype correlation, results in a diverse range of clinical manifestations, even in affected individuals within the same family. With recent therapeutic advancements in cholestasis treatment and the improved survival rates with liver transplantation (LT), many patients with ALGS survive into adulthood. Although LT is curative for liver disease secondary to ALGS, complications secondary to extrahepatic involvement remain problematic lifelong. This review is aimed at providing a comprehensive review of ALGS to adult clinicians who will take over the medical care of these patients following transition, with particular focus on certain aspects of the condition that require lifelong surveillance. We also provide a diagnostic framework for adult patients with suspected ALGS and highlight key aspects to consider when determining eligibility for LT in patients with this syndrome.

## Introduction

Alagille syndrome (ALGS) is a multisystem condition that includes cholestatic liver disease, cardiac, renal and vascular involvement, skeletal and ocular anomalies, and characteristic facial features. Following its first clinical description in 1969 [1], molecular studies have identified *JAGGED1 (JAG1)* and *NOTCH2* as the two disease-causing genes and mutations in these genes are inherited in an autosomal dominant fashion with incomplete penetrance [2–4]. This results in a highly variable disease phenotype with a wide array of organ involvement and disease severity [2, 4]. Although this is a developmental disorder, clinical features of ALGS may become apparent later in life, and therefore, ALGS should not be conceptualized as exclusively a disease of childhood. The majority of patients with ALGS are diagnosed during childhood, typically presenting with neonatal cholestasis and transition from pediatric to adult care with an established diagnosis. However, it is certainly possible that individuals with subtle or atypical features of ALGS may be missed in childhood and, therefore, this diagnosis can certainly be made for the first time in adults [5]. Approximately 90% of children with ALGS survive beyond the age of 18, including patients with and without cholestasis [6], so numerically, adults with ALGS are not ultra-rare. Since some features of ALGS can manifest or progress in adulthood and mutations are transmitted with autosomal dominant inheritance, it is crucial for adult health care practitioners to be aware of this condition. Historically hepatologists have led and coordinated the multidisciplinary care of children with ALGS, and this approach is likely also necessary in adulthood.

Given the paucity of data to guide the management of adults with ALGS, we provide a comprehensive review of the clinical phenotype of this syndrome, with a specific focus on features reported in adults with recommendations for surveillance and treatment.

## Features of Alagille syndrome and relevance in adulthood

### Hepatic involvement

The classic clinical feature of ALGS is neonatal cholestasis with high gamma-glutamyl transferase, which is present in 85% and is associated with pruritus in 74% of patients [6]. Complications secondary to cholestasis are burdensome in early childhood and include fat-soluble vitamin deficiency (FSVD), growth failure, and disfiguring xanthomas [7]. In the current era with readily available genetic testing, a liver biopsy is no longer necessary for the diagnosis of ALGS. However, paucity of intrahepatic bile ducts, though not pathognomonic for ALGS, remains the most characteristic histological feature of this disease and will be discussed further below.

As mentioned above, most adults with liver involvement due to ALGS are likely to have transitioned from childhood. There are limited data describing individuals diagnosed with ALGS as adults. The largest series of 8 patients (4 with *JAG1* and 4 with *NOTCH2* variants) were recently published from China [8], in which the median age at diagnosis was 24.5 years (range 16–56), and all patients had abnormal liver biochemistry and jaundice at presentation. Although the diagnosis was made in adulthood in this small series, three patients had a history of cholestasis of unknown etiology since childhood. This presence of an undiagnosed liver disease in childhood, whether it persists or not to adulthood, is a common observation in other case reports in the adult ALGS literature. [9–11]

### Hepatocellular carcinoma in ALGS

Hepatocellular carcinoma (HCC) is a rare but important complication of ALGS that can develop in childhood or adulthood. Original studies hypothesized its development as a consequence of persistent cholestasis [12]. However, further reports of HCC in ALGS patients without overt liver pathology, suggest a possible link between ALGS and HCC through interference with the Notch signaling pathway [5, 13, 14]. Aberrations within this highly conserved pathway have already been well described in other forms of solid organ malignancies such as pancreatic, breast, and ovarian cancer [15]. Within the liver, the Notch pathway plays a major role in hepatic cellular differentiation, which in the setting of progressive fibrosis, may result in HCC tumorigenesis and progression [16]. Further studies, are needed to support this novel theory.

The first report of HCC in ALGS was in 1986 by Ong et al., who described a 3-year-old child with HCC developing on a background of a cirrhotic liver secondary to ALGS [17]. Since then, a total of 21 cases in children and 14 cases in adults with ALGS and HCC have been described. The details of these cases are summarized in a recent report by Schindler et al. [5]. It is critical to emphasize that not all patients with HCC had pre-established cirrhosis. Among cases describing presence/absence of cirrhosis, 11% of children and the majority of adult cases (67%) had no evidence of cirrhosis [5, 9, 13, 14, 18–21]. In addition, 38% of patients had normal serum levels of alpha-fetoprotein (AFP) [5, 21–27], which further argues against the reliability of using AFP alone as a screening tool for HCC.

There are no HCC surveillance guidelines in ALGS. Previous reports suggest that the development of HCC in ALGS is unpredictable and that these tumors are resistant to chemotherapy, and difficult to treat with resection or LT [20, 28]. Thus, we recommend screening ALGS patients of all ages, regardless of the severity of hepatic involvement, with abdominal ultrasound (US) and serum AFP every 6 months. Contrast-enhanced cross-sectional abdominal imaging such as computed tomography (CT) or magnetic resonance imaging (MRI) should be reserved for patients with questionable lesions on US irrespective of serum AFP levels.

A common imaging finding that may be confused with HCC is the presence of hepatic regenerative nodules, particularly large-sized lesions (> 5 cm). These benign reactive lesions arise secondary to vascular abnormalities in cholestatic diseases causing preferential perfusion of certain areas of the liver leading to localized near-normal bile ducts in these regions [29]. Though not specific to ALGS, they are found in up to 30% of patients and do not require intervention. They can be distinguished from HCC radiologically by their proximity to the right portal vein (without vascular invasion), hypointensity on T2W and iso- to hyperintensity on T1W. Generally, the degree of nodular enhancement is comparable to that of the surrounding liver parenchyma [30]. A liver biopsy, is only required in situations where imaging studies fail to distinguish between these lesions [29].

### Cardiac involvement

Congenital heart disease is the most common extrahepatic feature of ALGS and is present in up to 94% of children with predominance of right-sided lesions [31]. Classic lesions found in ALGS patients include peripheral pulmonary artery stenosis (PPS) or hypoplasia (76%), and structural intracardiac anomalies such as teratology of Fallot (TOF) with or without pulmonary atresia (PA) in 12%. Left-sided lesions are far less common with supravalvular aortic stenosis occurring in 7% of patients. Complex heart disease is an important source of morbidity and mortality in childhood. Survival rates are poor in children with complex intracardiac lesions [32].

Adults with ALGS are likely to have already diagnosed and treated cardiac anomalies, or have subtle cardiac involvement. Since ALGS is a developmental disorder, there is no new-onset progressive cardiac disease after childhood. However, cardiac defects may be identified for the first time in adulthood, which if associated with liver involvement, should raise suspicion for ALGS. Finally, the hyperlipidemia associated with cholestatic ALGS is predominantly lipoprotein X which has no atherosclerotic consequences [7]. The implications of cardiac defects on LT candidacy will be discussed further below.

### Facial features

Facial dysmorphism is one of the most penetrant features of ALGS, particularly in JAG1 mutation-positive patients [33]. An inverted triangular face is typically described and is formed by a prominent forehead, deep-set eyes with hypertelorism, a straight nose with a bulbous tip, and a pointed chin. These features can correctly identify ALGS children in almost 80% by dysmorphologists [34]. Over time, however, facies change with less forehead dominance and further chin prominence and prognathism in adults, making it more difficult to identify, even by dysmorphologists in 67% of patients. Recognition of these features in adults, who may have other subtle signs of ALGS, may aid adult clinicians in their evaluation, particularly when presenting with a seemingly idiopathic disease involving multiple organ systems.

### Ocular features

Numerous ocular abnormalities have been described in ALGS. Within the anterior segment, posterior embryotoxon, which is of no visual consequence, remains the most common finding and is present in 51% of patients [6]. Notably, it is not specific to ALGS and may be present in approximately 22% of the general population and in other genetic syndromes [35, 36]. Additional commonly reported anterior segment findings include small corneal diameters and iris stromal hypoplasia [37]. Within the posterior segment, optic nerve head abnormalities, abnormally angulated vessels, and pigmentary retinopathy are characteristic of the fundus in ALGS. The ophthalmic spectrum of ALGS is much wider, however, with some lesions affecting vision in 73% of patients in a recent adult case series [38, 39]. This demands that all adult patients suspected of having ALGS should undergo a formal slit-lamp examination by an ophthalmologist familiar with ALGS-related ophthalmologic findings.

### Skeletal features

Butterfly vertebrae, secondary to sagittal clefting of the vertebral bodies, can be seen in 44% of ALGS patients, typically in the thoracic vertebrae [6]. They are of no clinical consequence not specific to ALGS, but are a helpful aid in the clinical diagnosis. Other anomalies such as radio-ulnar synostosis and rib abnormalities have also been described [40]. Pathological fractures, particularly of the lower extremities can occur in up to one third of patients without significant preceding trauma [41]. While fractures may be caused by persistent cholestasis and vitamin D deficiency, a recent study by Kindler et al. unveiled significant bone microarchitectural deficits using high resolution peripheral quantitative CT in children with ALGS in whom only 10% had low serum 25-hydoxyvitamin D levels, [42] suggesting that intrinsic bone defect may be secondary to impaired Notch signalling [43]. These abnormalities further reinforce recommendations for obtaining bone densitometry when patients are evaluated for LT and treatment of osteoporosis be initiated if indicated [44]. Due to the relative ease of detecting butterfly vertebrae, all adults suspected of having ALGS should have plain radiographs of the spine. Clinicians may also consider re-examining spinal images on historical cross-sectional three-dimensional imaging studies performed for other reasons.

### Renal features

Renal anomalies have been recognized as a disease-defining feature of ALGS and have, therefore, been incorporated in the syndrome’s clinical criteria [45]. Both structural and functional abnormalities have been reported in up to 40% of children with ALGS [46]. Among 73 *JAG1*-mutation positive children with renal involvement, dysplasia accounted for 59% of abnormalities, followed by renal tubular acidosis (9%), vesicoureteric reflux (8%), and obstructive uropathy (8%) [46]. Reno-vascular hypertension may also occur in 2–8% of patients [47]. End-stage renal disease progressing to renal transplantation is exceedingly rare in childhood.

Due to the absence of long-term studies, the prognosis and natural history of renal disease associated with ALGS is unclear [48]. However, there are numerous reports of familial chronic kidney disease and/or medically refractory hypertension in adults with ALGS (median age 38y, range 16–62), that require renal replacement therapy and/or renal transplantation [11, 49–57]. Although these cases do not represent the wide spectrum of renal phenotype associated with ALGS, it is possible that a small proportion of patients develop progressive renal disease. In cases of severe liver and renal disease, combined liver kidney transplantation has been successful in one adult with ALGS [53].

Worsening renal disease can take place at the time of transition from pediatric to adult care. Adult clinicians should evaluate patients’ kidney function with blood pressure measurement, renal US (with Doppler interrogation), measurement of serum electrolytes and creatinine, and urinary protein/creatinine ratio and electrolytes. These investigations are equally important to assess in the adult patient suspected of ALGS.

### Vascular features

Intracranial anomalies have been well described in the ALGS literature. Vascular anomalies, particularly ones causing intracranial hemorrhage (ICH), occur in 12–15% of patients and are responsible for the majority of non-cardiac deaths in 25–50% childhood cohort studies [32, 58–60]. The clinical presentation is quite variable, and although influenced by the location and severity of lesions, may range from asymptomatic silent infarcts to fatal spontaneous bleeding events. As reported by Emerick et al., cerebrovascular abnormalities were detected in 50% of asymptomatic, and virtually 100% of symptomatic children [32]. Reported intracranial lesions within the ALGS literature in children include absence/stenosis of the internal carotid artery (most commonly), basilar and middle cerebral artery aneurysms, epidural, subdural, and subarachnoid hemorrhage, and moyamoya disease [32, 58, 61]. Extracranial vascular anomalies (aside from PPS) involving the celiac trunk, renal, common hepatic, and superior mesenteric arteries, as well as aortic aneurysms and coarctation have been described in pediatric and adult ALGS literature [62–65].

Although no adult ALGS cohort studies exist, it is evident from limited research that the deleterious consequences of ALGS central nervous system (CNS) vasculopathy persist throughout adulthood (> 18 years). Review of the literature identifies 5 patients (age range 21–30, median 25) who experienced sudden subarachnoid hemorrhage (SAH) which lead to death in 40% [66–70]. Three of the bleeding events (60%) were caused by aneurysmal rupture involving the vertebrobasilar system [66, 67, 70], one involving the posterior communicating artery (PCA) [68], and one involving the internal carotid artery [69]. Two additional cases in 17-year-old patients, nearing transition to adult health care, developed SAH from ruptured PCA and superior cerebellar aneurysms have also been reported [71, 72]. These cases reveal that in contrast to the normal population, adults with ALGS are at a heightened risk of subarachnoid hemorrhage at a much younger age, with predominance of vertebrobasilar rather than anterior communicating artery aneurysms [73, 74]. In addition, symptomatic CNS infarcts have also been reported in adults. [64, 70]

There are currently no screening guidelines for intracranial vasculopathy in ALGS. However, it has been our practice to screen for these lesions with brain MRI and angiography (MRA) at 8 years of age, and prior to major surgeries. We also perform head imaging emergently in the presence of neurological symptoms regardless of age. In the adult population, screening should be performed in the following settings: (1) soon after transition to adult care for known ALGS patients if screening had not been previously done, (2) for a patient suspected to have ALGS, (3) prior to any major surgeries in patients with an established ALGS diagnosis, and (4) in the presence of neurological symptoms emergently for the known ALGS patient. Management of detectable lesions should be guided by a neurosurgeon and/or neurointerventionalist. The frequency of repeat imaging when a study is normal is unknown as this has not been systematically studied.

### Additional features

An increased risk of bleeding events has been recognized in patients with ALGS, even in the absence of vascular anomalies, trauma, or coagulopathy [75]. As reported by Lykavieris et al., 22% of ALGS patients without liver failure developed spontaneous bleeding, which was fatal in eight cases. Notably, only one death was due to ICH. The exact pathophysiology of bleeding diathesis is unknown but may be due to hemostatic and angiogenic dysfunction secondary to abnormal Notch signaling. Adult clinicians should be aware of bleeding tendency in ALGS individuals, particularly when undergoing a liver biopsy or LT.

Table 1 provides a comprehensive list of diagnostic procedures suggested for individuals suspected to have ALGS. A summary of recommended surveillance in adults with ALGS is highlighted in Table 2.

**Table 1:** Recommended investigations for adults with suspected ALGS (including index cases and patients referred for family screening)

	Body system	Work up
1	Hepatobiliary	a. Serum biochemistry with ALT, AST, ALP, GGT, total cholesterol, serum bile acids b. Hepatobiliary US with Doppler interrogation (if reduced visibility or a suspicious lesion is identified, refer to Table 2) c. Consider liver biopsy in atypical cases and/or when concomitant liver disease is suspected
2	Cardiac	Transthoracic echocardiogram
3	Eye	Formal slit-lamp examination
4	Skeletal	Plain X-ray of the spine or re-examining previous cross-sectional images
5	Face	Clinical examination for ALGS facies
6	Renal	a. Blood pressure measurement b. Serum electrolytes and creatinine c. Urinary electrolytes, urinalysis, and protein/creatinine ratio d. Renal US with Doppler interrogation
7	Cerebrovascular	Brain MRI/MRA
8	Genetics	*JAG1* and *NOTCH2* mutational analysis

*ALGS* Alagille syndrome, *ALT* alanine aminotransferase, *AST* aspartate aminotransferase, *ALP* alkaline phosphatase, *GGT* gamma-glutamyl transferase, *US* ultrasonography, *MRI* magnetic resonance imaging, *MRA* magnetic resonance angiography, *JAG1* Jagged1

**Table 2:** Recommended surveillance for adult patients with ALGS regardless of age at diagnosis

	Issue	Work up/management	Timing
1	HCC^†^	a. Hepatobiliary US and serum AFP regardless of presence/absence of hepatic involvement	Every 6 months
		b. Contrast-enhanced CT or multiphasic MRI for patients with reduced visibility on US and/or suspicious lesions ± liver biopsy	As indicated
2	Renal disease progression	a. Blood pressure measurement b. Serum electrolytes and creatinine c. Urinary electrolytes, urinalysis, and protein/creatinine ratio	Annual
3	Cerebrovascular bleeding	Brain MRI/MRA	At transition to adult care (if not previously done) Prior to major surgeries Presence of neurological symptoms
4	Pregnancy/childbirth-related complications	a. Pre-conception counselling b. Offer prenatal genetic testing c. Referral to a high-risk pregnancy specialist and obstetrical anaesthetist d. Endoscopic variceal screening as indicated (see text) e. Ensure strict adherence to fat-soluble vitamins if cholestatic f. Detailed fetal US as indicated (see text)	

*ALGS* Alagille syndrome, *HCC* hepatocellular carcinoma, *US* ultrasonography, *AFP* alpha-fetoprotein, *CT* computed tomography, *MRI* magnetic resonance imaging, *MRA*, magnetic resonance angiography

^†^Applies to ALGS individuals with native liver only

## Alagille syndrome and pregnancy

### Pregnancy-related complications

Pregnancy can pose significant risks to expectant females with ALGS and the fetus. Physiological increase in cardiac output due to splanchnic and portal compression by the growing uterus may worsen portal hypertension in pregnancy, which has been linked to increase in variceal bleeding during pregnancy, higher rates of prematurity, and spontaneous early abortion [76, 77]. Similarly, hemodynamic changes associated with pregnancy may cause cardiac function to deteriorate, particularly in patients with pulmonary hypertension due to PPS [33]. In addition, ALGS patients with cerebral aneurysms are at increased risk of aneurysmal rupture/bleeding with repeated Valsalva maneuvers associated with spontaneous vaginal delivery [78].

For these reasons (and to minimize maternal efforts), delivery via cesarean section or assisted vaginal delivery (forceps or vacuum) has been suggested as the delivery route of choice specifically in pregnant ALGS patients with intracranial vasculopathy, clinically significant portal hypertension, and severe cardiac disease [78, 79]. Unassisted vaginal delivery however, has been successful in ALGS expectant women without these issues [80, 81]. In fact, recent guidelines advocate for the mode of delivery in pregnant women with cirrhosis to be solely guided by obstetric indications [82]. It is recommended for such a delivery to be performed in a centre where there is rapid access to an endoscopist. Pre-conception endoscopic variceal screening (or in the second trimester if it has not been conducted in the preceding year) is advised to lower the risk of variceal hemorrhage in pregnant women with cirrhosis [82]. The management of varices is similar to those who are not pregnant [83].

Maternal cholestasis (which can worsen due to progestogenic biliary stasis) has been associated with increased risk of fetal distress with meconium-stained liquor, preterm delivery, and sudden fetal death close to term [80, 84]. Furthermore, FSVD in pregnancy can lead to numerous consequences such as vitamin K deficiency bleeding in the newborn, maxillary hypoplasia, fetal hypocalcemia, intrauterine growth restriction (IUGR), and hypoproteinemia [85]. Additionally, coagulopathy associated with vitamin K deficiency may render neuraxial anesthesia unsafe [80]. As such, it is essential that cholestasis be addressed and treated appropriately prior to embarking on pregnancy, with particular stress placed on adherence to fat-soluble vitamin supplementation before, during, and after pregnancy.

### Prenatal features of ALGS

ALGS is inherited from a parent in 40% of cases and arises de novo in the remaining 60% [86, 87]. Detailed fetal ultrasonography in the setting of ALGS is important to identify in-utero features of ALGS in cases where one parent is affected, especially if prenatal genetic testing was not performed, and to evaluate the severity of cardiac defects if ALGS was genetically confirmed in the fetus.

Commonly reported prenatal features of ALGS from eight case reports include IUGR in 75% of cases, cardiac anomalies (three cases, TOF in two and severe pulmonary stenosis in one), and hemivertebrae and two-vessel cord in two cases [78–81, 88, 89]. Other reported features include severe lumbosacral kyphoscoliosis, absent gallbladder, prominent chin, and prominent stomach with ascites [80, 90]. Fetuses suspected of having ALGS should receive detailed cardiac imaging (fetal echocardiography or MRI). If critical cardiac lesions are detected, delivery should take place at a tertiary level center with access to multidisciplinary services (neonatal intensivist, cardiologist, cardiac surgeon, gastroenterologist).

### Pre-conception counseling

Pregnancy with ALGS carries a significant burden on parents and the fetus, and parents need to be well-informed of the risks involved (as discussed above). Pre-conception counseling should explain the 50% risk for the fetus to inherit either parent’s mutation. However, due to the absence of genotype–phenotype correlations, it is impossible to predict disease severity in an affected fetus. If pregnancy is pursued, prenatal genetic testing should be offered. This can include non-invasive cell-free fetal DNA derived from maternal plasma that utilizes single-gene disorders (as part of non-invasive prenatal testing in the first trimester), or conventional interventions such as amniocentesis or chorionic villous sampling [91]. Alternatively, with the advent of assistive reproductive technology, preimplantation genetic diagnosis has been successful in diagnosing ALGS and could be considered. [92–94]

## Pathophysiology of Alagille syndrome

**Fig. 1:**
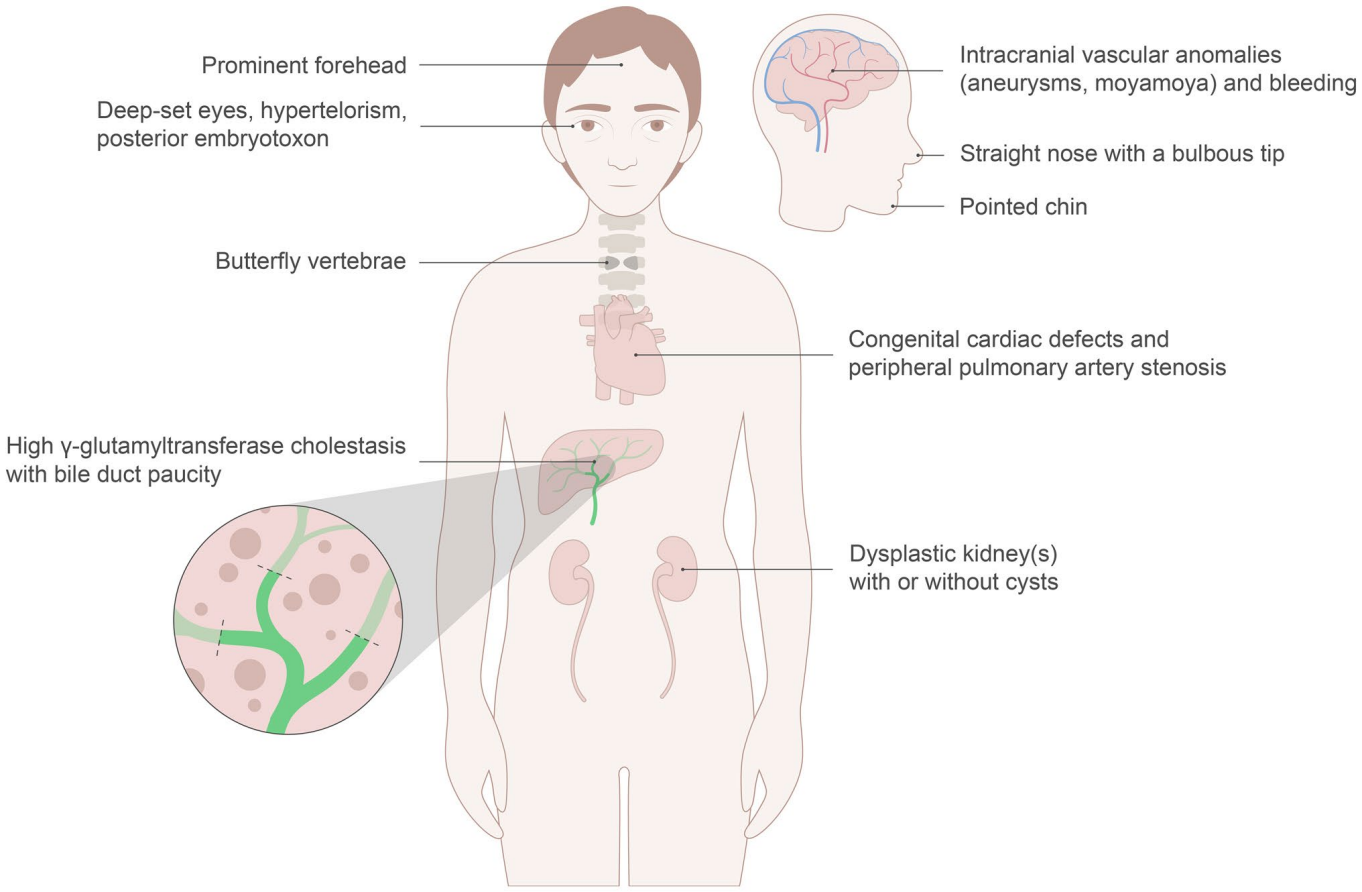
Spectrum of clinical features in Alagille syndrome

ALGS occurs as a result of impaired signaling within the highly conserved Notch signaling pathway, which is comprised of ligands that include *JAG1*, and four Notch receptors that include *NOTCH2* [95]. This pathway plays a crucial role in cell–cell communication, cell fate determination, and normal embryological morphogenesis [96]. Several human and murine studies have confirmed that *JAG1* and *NOTCH2* are highly expressed in organs typically affected in ALGS including the liver, heart, skeleton, eye, face, and kidney (Fig. 1) [97]. Perhaps most importantly in the context of cholestasis in ALGS, is the clear evidence from mouse studies that *JAG1-NOTCH2* activation is essential for the biliary development and tubular morphogenesis [98, 99]. The presence of bile duct paucity in *JAG1*-mutation positive ALGS patients further supports the significant role of the Notch signaling pathway in intrahepatic bile duct development [100, 101].

## Diagnosis of Alagille syndrome in adults

### The diagnostic criteria of ALGS

If a pathogenic variant is identified in *JAG1* or *NOTCH2*, the presence of at least one characteristic clinical feature is sufficient for a diagnosis of ALGS (Table 3) [45]. It should be noted that very few NOTCH2 variants have been described in the literature and, therefore, determining the pathogenicity of these variants is not straightforward. Adults often undergo targeted genetic screening after a diagnosis and mutation has already been identified in the family, typically their child. The clinical diagnosis of ALGS requires at least three characteristic clinical features (or two if there is a family history in a first-degree relative). Some clinical features associated with ALGS such as butterfly vertebrae or cerebral aneurysms are not unique to this disorder so caution must be applied when making a clinical diagnosis in atypical or subtle cases—and in these scenarios, particularly, a molecular diagnosis should be sought where possible.

**Table 3:** Diagnostic Criteria for ALGS

Pathogenic variant in *JAG1* or *NOTCH2*	Family history of ALGS	Number of clinical features required^†^
Identified	Present	Any or none
Identified	None (proband)	At least 1
Not identified or not screened for	Present	2 or more
Not identified or not screened for	None (proband)	3 or more

*ALGS* Alagille syndrome, *JAG1* Jagged1

^†^Clinical features include paucity of bile ducts and/or cholestasis, cardiac, renal, ocular, or skeletal manifestations, structural vascular anomalies or events, and/or characteristic facial features

### Liver histopathology

As discussed previously, a liver biopsy is no longer required for diagnosis. However, it may be necessary in cases where mutational analysis is not possible or unrevealing and also especially in adults where co-existing liver diseases may need to be considered such as fatty liver or viral hepatitis. Bile duct paucity remains the hallmark histological feature of ALGS. The liver biopsy specimen must have at least six to ten portal tracts for a formal assessment of paucity, which is established if the ratio of interlobular ducts to portal tracts is less than 0.9 [102, 103]. A recent multicentered international study led by The Global Alagille Alliance Study Group (GALA), provides the largest review of histopathology reports in ALGS to date [6]. Bile duct paucity was found in 70% of ALGS patients with cholestasis (*n = *421/604) and reaching up to 87% in children above the age of 2. This is a well-known phenomenon in ALGS in which the prevalence of bile duct paucity identification considerably rises with age. A small minority of biopsies performed in young infants may show features of biliary obstruction (e.g., bile plugs and biliary proliferation) and giant cell transformation. This pattern, however, is unlikely to be seen in adults as these features substantially decrease overtime in early childhood.

Although bile duct paucity has been identified in almost all adult patients with ALGS and hepatic involvement reported in the literature [8–10, 54, 104, 105], it can also be found in patients with primary sclerosing cholangitis, primary biliary cholangitis, cystic fibrosis liver disease, alpha-1 antitrypsin deficiency, and drug induced liver injury [106, 107]. Caution must, therefore, be exercised when relying solely on liver histopathology to diagnose ALGS and further supports the importance of utilizing genetic testing (when feasible).

### Genetic testing

To date, 694 pathogenic variants in *JAG1*, and 19 pathogenic variants in *NOTCH2* have been reported in individuals with clinical features of ALGS [108]. Molecular testing for mutations within these genes establishes the diagnosis in 97% of cases. *JAG1* gene sequencing and large deletion/duplication analysis identifies a pathogenic mutation in 94% of cases, and *NOTCH2* sequencing identifies an additional 2.5% [108, 109]. Although testing may be carried out in a step-wise approach, it is more efficient to test both genes simultaneously using comprehensive cholestasis whole exome sequencing panels, which have captured ALGS in adults among other causes of genetic cholestasis [110].

Finally, although there are a very few patients described with pathogenic *NOTCH2* variants to date, it is notable that compared to their *JAG1* counterparts, those with *NOTCH2* variants appear to be less likely to have facial anomalies (52 vs 89%), cardiac anomalies (38 vs 98%), posterior embryotoxon (13 vs 52%), or butterfly vertebrae (0 vs 43%), suggesting that *NOTCH2*-ALGS patients have lower penetrance of extrahepatic features. [111]

## Management of Alagille syndrome

There are no data to support specific strategies to treat ALGS-related liver disease in adults. Although the management of cholestasis is not expected to be different to that in children, the consideration of comorbid conditions in adults do need consideration.

### Nutritional and medical management of cholestasis

Supporting nutrition is at the cornerstone of any cholestatic liver disease and is even more relevant in the setting of ALGS where many patients have cardiac disease resulting in increased metabolic demands. The focus remains on meeting energy needs, supplementation with passively absorbed calorie-dense medium-chain triglycerides and fat-soluble vitamins, and treating mineral bone disease [112]. As malnutrition has been linked to poor outcome post-LT, nutritional assessment and optimization by an experienced dieticians is invaluable in the evaluation of ALGS patients for LT candidacy [44].

Conventional pharmacological management of cholestatic pruritus follows a step-wise approach and is associated with variable success. In children, this involves ursodeoxycholic acid, antihistamines, rifampicin and sometimes naltrexone. However, additional agents such as fibrates which are commonplace in the management of adult cholestasis may also be helpful, though their use in ALGS has not been reported. A novel treatment for pruritus in ALGS are ileal bile acid transporter (IBAT) inhibitors. These drugs pharmacologically interrupt the enterohepatic circulation (EHC) of bile acids at the level of ileal enterocytes, which reduces bile acid return to the liver and reduction of total bile acid pool size [113, 114]. Maralixibat (MRX), an IBAT inhibitor taken orally, has been shown to be efficacious in three placebo-controlled multicentered randomized phase IIb studies with open label extension in patients (aged up to 18 years at study enrollment) with cholestasis due to ALGS [115, 116]. Collectively, MRX-treated patients had significantly improved pruritus, xanthomas, growth, fatigue, quality of life, and reductions in serum bile acids and cholesterol.

These studies show that MRX was well tolerated with mild gastrointestinal side effects and elevated alanine aminotransferase occurring in only a small number of patients. Unlike other methods of EHC interruption such as bile acid resins or surgical biliary diversion, fat-soluble vitamin deficiencies did not worsen during the extension period of all studies. Furthermore, a recent press release revealed topline data from the ASSERT study, a pediatric phase III trial evaluating the efficacy of odevixibat (Bylvay^®^) in patients with ALGS. Cholestatic children and adolescents (0–17 years of age) with ALGS on odevixibat experienced statistically significant improvement of pruritus and reduction of serum bile acids compared to placebo, with comparable rates of drug-related diarrhea. Odevixibat has already been approved by the U.S Food and Drug Administration (FDA) for the treatment of pruritus in patients with progressive familial intrahepatic cholestasis ≥ 3 months of age, and is currently pending submission of regulatory filing in the US for use in ALGS. Ultimately, these studies show that IBAT inhibitors are promising novel agents that can be added to the arsenal of antipruritic/cholestatic agents in ALGS. MRX (Livmarli^®^) has been approved by the U.S FDA in 2021 for treatment of cholestatic pruritus in patients with ALGS aged 1 year and older. In fact, data on file at Mirum Pharmaceuticals (unpublished data) show that seven MRX-treated patients in the aforementioned clinical trials were adults (≥ 17 years of age), and most have continued treatment beyond study end dates. Therefore, MRX can be considered as a treatment option in adults with pruritus refractory to other agents.

### Surgical management of cholestasis

An external biliary diversion (EBD) procedure serves as a surgical interruption of the EHC typically by the use of a jejunal conduit to drain the gallbladder externally. It has been shown to be effective in treating severe pruritus in ALGS with improved xanthomas and reduced serum cholesterol [117]. As the peak of cholestasis in ALGS usually takes place in childhood, some patients may have undergone an EBD procedure prior to transition. It should be noted that stoma losses may contribute to severe dehydration and electrolyte imbalances in gastroenteritis. Overall, EBD may be considered in the rare event of progressive pruritus in young adults with ALGS though IBAT inhibition is easier and more palatable to patients.

## Liver transplantation in Alagille syndrome

The outcome of liver disease in children with ALGS with respect to liver transplantation was previously regarded as favorable from single-center studies. However, the aforementioned GALA study enabled the capture of real-world natural history data [6]. Among 1184 children with ALGS and cholestasis, 349 (29%) underwent LT at a median age of 2.8 years, with the majority (~ 70%) of transplants performed before the age of 5. Moreover, the cumulative incidence of LT in the presence of competing events (death without LT and native liver survival) at 5, 10, and 18 years was 27, 38, and 50%, respectively. More relevant to the adult hepatologist, only 40% of patients with cholestasis in infancy survived with their native liver to the age of 18 years and 70% of these had clinically evident portal hypertension. Adult clinicians should be aware of the heightened risk of hepatic decompensation at the time of transition to adult care, which may be fatal [118].

### Liver transplantation in adults

Approximately, 10% of ALGS individuals require LT in adulthood [119]. A report by Arnon et al. from United Network for Organ Sharing (UNOS) described the outcome of 44 adult LT patients with ALGS (median age 30 years) over a 21-year period, which accounted for only 0.05% of all combined adult LT cases (outcomes described below) [119]. As the UNOS database does not capture extrahepatic features, it can be speculated that ALGS patients survived into adulthood, because they did not have significant cardiac anomalies.

The overall indications for LT in adults are similar to children with ALGS [119, 120]. However, it is likely that more adults than children are transplanted for end-stage liver disease, portal hypertension and associated complications rather than pruritus and persistent cholestasis. The GALA study revealed that more than two-thirds of patients developed portal hypertension by 18 years of age, which had sharply risen from 39% at the age of 10. Portal hypertension was in fact the main indication for LT among older ALGS recipients in this large cohort [6].

As described by Arnon et al., compared to children (*n = *507), adults (*n = *44) had lower serum albumin and higher serum creatinine which is reflected in the higher MELD/PELD (22 vs 17) [119]. Furthermore, adults had higher rates of hepatic encephalopathy at the time of LT (23% vs 5%). These findings suggest higher rates of hepatic synthetic dysfunction in adults and highlights the progressive nature of renal disease over time [119]. The outcomes of LT in adult ALGS recipients were good and are comparable to the overall pooled adult LT survival data, with 96 and 91% patient survival at 1 and 5 years, and 84 and 80% graft survival at 1 and 5 years, respectively. These outcome metrics were better to those of children, though not statistically significant. In addition, death within 30 days post-LT and graft loss within 14 days were higher in children, for which serum creatinine and cold ischemia time were significant predictors of these events. No predictors of death or graft loss were detected in adults, surprisingly not even creatinine. Long-term follow-up revealed that adults had lower rates of vascular thrombosis (8 vs 25%) and primary non-function (8 vs 21%) than children, resulting in graft loss and death in 16% of adults and 20% of children. Cardiac disease post-LT was fatal in 10% of children but none in adults. These higher rates of complications post-LT in ALGS children, however, no longer seem to hold true in more contemporary pediatric studies, likely due to better understanding of the systemic involvement in ALGS leading to improved care and outcomes post-LT [121].

## Impact of Alagille syndrome on liver transplantation

Table 4 outlines key issues that should be considered by the transplant hepatologist when determining LT eligibility for an adult ALGS patient.

**Table 4:** Recommendations for The Adult Transplant Hepatologist When Evaluating an ALGS Patient for LT Eligibility

	Aspect	Recommendation
1	Living-related donor selection	Screen potential living-related donors with mutation analysis of *JAG1* and *NOTCH2* genes (for both liver and kidney transplant candidates)
2	Cardiac reserve evaluation	a. Screen all ALGS patients with stress echocardiography b. Consider cardiac catheterization with pharmacological stress testing in selected cases as clinically indicated c. Surgical correction of severe anomalies pre-LT may be considered
3	Renal function preservation	All ALGS patients should receive a renal-sparing immunosuppression regimen post-LT
4	Cerebrovascular bleeding	Screen all ALGS patients for vascular anomalies with brain MRI/MRA
5	Intra-abdominal arterial anomalies	Screen all ALGS patients pre-LT for visceral vasculopathy with abdominal triphasic CT scan

*ALGS* Alagille Syndrome, *LT* liver transplantation, *JAG1* Jagged1, *MRI* magnetic resonance imaging, *MRA* magnetic resonance arteriogram, *CT* computed tomography

### Living-related donor screening

Solid organ living-related transplantation is becoming more frequent with ongoing scarcities in deceased organ donors. This has significant implications in ALGS since 40% of patients inherit the disease from one of their parents who may be considered for living donation but may not have overt clinical signs or symptoms of ALGS [122]. Previous reports have described the unexpected intraoperative discovery of bile duct paucity and hypoplasia in parents of children with ALGS despite no evidence of biochemical or radiological liver disease during the donor evaluation process, leading to termination of living-related LT [122]. Therefore, targeted sequencing for *JAG1* and *NOTCH2* variants is recommended for all potential living-related donors in ALGS including liver and kidney transplant candidates. If a variant is not known in the family, then living-related donors for an ALGS patient should undergo an extensive clinical evaluation, including imaging for intra-abdominal vascular anomalies and liver biopsy.

### Evaluation of cardiac function prior to LT listing

Optimization of cardiac function prior to LT listing is paramount to avoid morbidities associated with transplantation and cardiac complications in the immediate post-operative period. Patients with PPS/hypoplasia and right ventricular outflow tract obstruction may develop pulmonary hypertension over time. This can significantly worsen during the anhepatic phase of LT surgery, and lead to marked increase in preload and cardiac failure. To address this, cardiac catheterization with a dobutamine challenge test has been adopted in some centres to simulate LT surgery hemodynamics in ALGS [123]. Cardiac reserve is considered sufficient if cardiac output rises by 40% and right ventricular/aortic pressure ratio is ≤ 0.5. In another study, however, a ratio > 0.5 was not predictive of early death post-LT, nor was it a contraindication for LT [124]. Regardless, in instances where significant cardiac disease is present, catheter-based or open surgical treatment should be sought in consultation with a cardiovascular surgeon, whilst balancing risks and benefit of LT and/or corrective cardiac surgery [125].

There are no ALGS-specific cardiac guidelines for adults, nonetheless the 2015 liver transplantation clinical practice guidelines from the European Association for the Study of the Liver recommend electrocardiogram and transthoracic echocardiography for all LT candidates, with stress testing reserved for patients with multiple risk factors or over 50 years of age [126]. The 2013 American Association for The Study of Liver Diseases practice guideline for evaluation of liver transplantation in adults, recommends cardiac evaluation for all adult LT candidates with stress echocardiography as an initial screening tool, and cardiac catheterization be considered as clinically indicated [44]. The 2012 American Heart Association and American College of Cardiology consensus statement on cardiac disease evaluation among liver transplant candidates share similar guidance [127].

### Surgical technical variants for intra-abdominal vasculopathy

Screening for intra-abdominal vascular anomalies is crucial to allow planning for operative surgical vascular modifications at the time of LT, since virtually all of these anomalies are asymptomatic. Kohaut et al. reported that among 55 pediatric ALGS cases, almost 64% underwent aortic conduit reconstruction, which was associated with a significant reduction in the incidence of hepatic artery thrombosis compared to patients who underwent standard arterial anastomosis (6 vs 35%) [62]. Although this may be less problematic in adults given the larger-sized vessels, it remains essential for ALGS patients considered for LT undergo cross-sectional abdominal imaging (triphasic CT scan) to guide surgical planning and for proper counseling of LT risks prior to listing.

### Preservation of renal function post-LT

There are no formal studies evaluating long-term renal function post-LT in adults with ALGS. In children, however, it has been well described that ALGS individuals have higher rates of serum creatinine and renal insufficiency at the time of LT, with at least 25% of patients having an estimated glomerular filtration rate less than 80 ml/min/1.73m^2^ (i.e., grade 2 chronic kidney disease) as observed by Black et al. [121] Other studies, in fact, revealed that that renal insufficiency significantly worsens post-LT in 22% when compared to only 8% of their BA counterparts [128]. As discussed previously, renal disease is progressive in some ALGS patients, which is evident by higher rates of renal insufficiency in adults ALGS patients compared to children at the time of LT, and the substantial reports of renal transplantation in adults [119]. As a result, it is prudent to adopt a renal-sparing immunosuppression regimen post-LT, with delayed introduction of calcineurin inhibitors to avoid further strain on the developmentally abnormal kidneys.

## Conclusions

ALGS is a fascinating disease with multiple manifestation involving major organ systems spanning from infancy to adulthood. The significant intrafamilial heterogeneity and lack of genotype–phenotype association makes ALGS an unpredictable disease that poses a challenge to clinicians of all specialties providing care for these patients across all ages. The management of cholestatic pruritus in ALGS has advanced with the advent of IBAT inhibitors, which has proved to be a life-changing treatment for many children. However, there is still a need for therapies that may target bile duct paucity and for progress in the management of extrahepatic disease secondary to ALGS. With the high rate of survival of these patients into adulthood, both with and without native liver, deepening our understanding of the broad phenotype of ALGS is necessary to deliver personalized care. The responsibility to coordinate the complex care of patients with ALGS continues to rest with pediatric and adult hepatologists alike.

## Abbreviations

ALGS: Alagille syndrome
LT: Liver transplantation
IBAT: Intestinal Bile Acid Transport
JAG1: Jagged1
FSVD: Fat-soluble vitamin deficiency
HCC: Hepatocellular carcinoma
AFP: Alpha-fetoprotein
US: Ultrasonography
CT: Computed tomography
MRI: Magnetic resonance imaging
PPS: Peripheral pulmonary artery stenosis
TOF: Tetralogy of Fallot
PA: Pulmonary atresia
ICH: Intracranial hemorrhage
CNS: Central nervous system
SAH: Subarachnoid hemorrhage
PCA: Posterior communicating artery
MRA: Magnetic resonance angiography
IUGR: Intrauterine growth restriction
GALA: The Global Alagille Alliance Study Group
EHC: Enterohepatic circulation
MRX: Maralixibat
FDA: Food and Drug Administration
EBD: External biliary diversion
BA: Biliary atresia
UNOS: United Network for Organ Sharing
